# Irinotecan-Loaded Vaterite Microspheres for Drug Delivery: Drug Release and Dissolution Kinetics and Mechanism in an Aqueous Solution and Human Serum

**DOI:** 10.1021/acs.langmuir.6c01242

**Published:** 2026-05-27

**Authors:** Morgan P. Milner, Hugo A. Saint, Jake M. Yang, Christopher C. M. Neumann, Katharina Wansch, Richard G. Compton

**Affiliations:** † Physical and Theoretical Chemistry Laboratory, Department of Chemistry, 6396 University of Oxford, Oxford OX1 3QZ, Great Britain; ‡ St John’s College, St Giles, Oxford OX1 3JP, Great Britain; § Centre for Sustainable Materials Processing, School of Chemistry, University of Leicester, Leicester LE1 7RH, Great Britain; ∥ Department of Hematology, Oncology and Tumor Immunology, Charité-Universitätsmedizin Berlin, Freie Universität Berlin, Humboldt-Universität zu Berlin, Berlin Institute of Health, Charitéplatz 1, D-10117 Berlin, Germany

## Abstract

The coprecipitation synthesis of calcium carbonate vaterite microspheres loaded with chemotherapeutic drug irinotecan is reported. High drug loading comparable to the best reported for other drugs in porous vaterite is achieved, and characterization confirms that the characteristic spherical morphology of vaterite is not disrupted by the presence of irinotecan. UV–vis spectroscopy demonstrates the release of the drug over time, and the latter, upon comparison with optical microscopy dissolution data, shows that drug release correlates with the time scale of dissolution of the vaterite host. To model application in a drug delivery context, the dissolution of unloaded vaterite and irinotecan-loaded vaterite in human serum and diluted human serum is investigated, revealing inhibition of dissolution by human serum that occurs in two distinct serum concentration-dependent ranges. Furthermore, irinotecan-loaded vaterite is tested in vitro on two human pancreatic cancer cell lines, MiaPaCa-2 and Capan-1, demonstrating a therapeutic effect in both cases.

## Graphical abstract

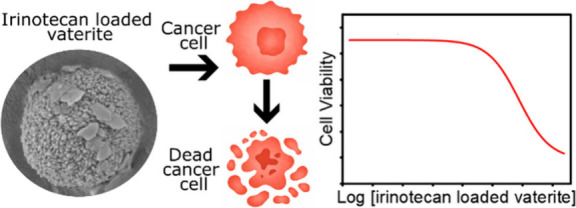

## Introduction

Conventional cancer treatments, such as surgery, radiotherapy and chemotherapy, undoubtedly provide significant therapeutic benefits; however, their potential drawbacks - particularly the risk of damage to healthy tissue - have driven substantial interest in the development of new, tumor-specific and innovative treatment approaches. A promising strategy that has emerged is the use of targeted drug delivery systems, which enable the controlled release of drug molecules at the site of cancer, thereby enhancing therapeutic efficacy and reducing side effects.^1,2^ The tumor microenvironment is characterized by biochemical features, such as low pH and altered enzyme activity, as well as pathological differences including abnormal vascular structure.^3–5^ Modern drug delivery systems, such as inorganic micro- and nanoparticles, liposomes, and dendrimers, take advantage of these conditions to achieve more effective and targeted drug delivery.^6–8^ Other benefits of these drug delivery systems include improved kinetic solubility and cellular uptake of therapeutics, protection of drugs from harsh biological environments to extend circulation time, and the codelivery of therapeutic and diagnostic agents to enhance treatment efficacy and enable real-time monitoring.^6^

In recent years, inorganic materials have been noted as promising carriers in drug delivery systems due to their ability to load and release drugs while maintaining structural stability, offering tunable degradation rates, and providing higher drug-loading capacity compared with organic carriers.^9–11^ Porous vaterite has been widely used as a carrier in drug delivery systems because its well-developed internal structure enables the incorporation of both low-molecular-weight compounds and macromolecules.^12,13^ Moreover, its enhanced dissolution under acidic conditions supports a pH-responsive release mechanism, enabling targeted drug delivery in relatively acidic extracellular microenvironments, most notably those surrounding tumors, as explored in this work.^14,15^ Recent work has shown vaterite can be employed as a ‘sacrificial matrix’, where biomolecules are loaded using either an adsorption or coprecipitation route, followed by the vaterite surface being coated with a polymer shell and the core being dissolved.^16^ In other cases, the core is retained and used together with the shell to increase resistance to external influences. Some systems have also been proposed that do not require shell formation, particularly when parenteral administration is intended, as this avoids the acidic environment of the stomach in which vaterite quickly dissolves.

Fundamental studies of the dissolution behavior and structural stability of vaterite in different media are essential for both modeling and evaluating its effectiveness as a drug delivery system. Recent advances have employed optical microscopy to investigate the dissolution of individual vaterite (and calcite) particles, as well as biomineralized coccoliths and coccolithophore cells.^17–21^ This approach enables investigation within a well-defined (‘sphere on a plate’) diffusive mass-transport regime, allowing the extraction of kinetic and thermodynamic insights and parameters. It has been shown that the dissolution of both spherulitic monomer and aggregated (pure) vaterite particles occurs under thermodynamic control, with the rate governed by the solubility product and the rate of diffusion away of the dissolved vaterite from the dissolving interface.^17,18,22^ The synthesis of vaterite/hydroxyapatite core–shell particles has also been developed, exhibiting a dissolution process that proceeds in two thermodynamically controlled stages corresponding to the dissolution of the shell and of the core.^23^ In addition, the effect of magnesium adsorption on vaterite dissolution and its conversion to calcite has been investigated.^24^

Irinotecan shows strong antitumor activity and is widely used in the treatment of various cancers, including pancreatic ductal adenocarcinoma. Its associated side effects, including myelosuppression and diarrhea, constitute its dose-limiting toxicity, meaning that its incorporation into drug delivery systems could be highly beneficial in reducing exposure to healthy cells.^25^ Incorporation of irinotecan into advanced drug delivery systems has previously shown promising results in increasing antitumoral activity. In particular, a recent phase III clinical trial demonstrated that the liposomal formulation of irinotecan achieves reduced clearance, prolonged half-life, and enhanced tumor accumulation compared to the conventional formulation, supporting its improved therapeutic profile.^26,27^ Most importantly, this trial showed the most substantial survival improvements for metastatic pancreatic ductal adenocarcinoma in over a decade, highlighting the transformative potential of advanced drug delivery systems enhancing irinotecan’s efficacy.^27^ In the work reported here, irinotecan is novelly incorporated into vaterite, and its dissolution behavior in aqueous conditions, as well as in pure and diluted human serum, is presented. Furthermore, the antitumoral activity of irinotecan-loaded vaterite as well as conventional irinotecan is tested in human pancreatic cancer cell lines MiaPaca-2 and Capan-1.

## Experimental Section

Ultrapure deionized (DI) water (resistivity close to 18.2 MΩ·cm at 298 K; pH ≈ 6.7), obtained from a Milli-Q system, was used in all experiments except for cell culture and pharmacotyping studies. The pH was measured using a Hanna Instruments HI5221 pH meter equipped with a HI1131B glass refillable probe.

Note that there are no unexpected, new or significant hazards associated with the presented work.

### Particle Synthesis

Following the method of Milner et al., spherical vaterite microparticles were synthesized by adding 3 mL of 1 M Na_2_CO_3_ to a solution of 3 mL of 1 M CaCl_2_·2H_2_O and 9 mL of deionized water.^17,18^ The mixture was stirred at 650 rpm for 45 s and maintained at 25 °C for 15 min. The particles were collected by centrifugation at 2500 g for 1 min, washed once with deionized water, and then suspended in 1–2 mL of methanol, which was allowed to evaporate at room temperature to obtain dried vaterite.

Irinotecan-loaded vaterite particles were synthesized using a similar procedure, maintaining the same calcium and carbonate ion concentrations and stirring conditions, with the addition of irinotecan hydrochloride and minor modifications. Solutions of 400 mM CaCl_2_·2H_2_O containing 5 mM irinotecan hydrochloride (7.5 mL) and 400 mM Na_2_CO_3_ containing 5 mM irinotecan hydrochloride (7.5 mL) were mixed, followed by stirring for 45 s and a subsequent 15 min period without agitation. After centrifugation, the supernatant was retained for analysis, and the particles were isolated through three washing steps consisting of resuspension in 15 mL of water, centrifugation, and particle separation. Finally, the particles were resuspended in a small volume (∼0.5 mL) of water and dried in an oven at 80 °C for 1 h.

CaCl_2_·2H_2_O (≥99%, Sigma-Aldrich), Na_2_CO_3_ (99.6%, Acros Organics) and Irinotecan Hydrochloride (98.0%, Fluorochem) were used, and separations were performed using an Eppendorf 5702 centrifuge.

### Characterization

For SEM analysis, particles were deposited onto carbon tape and sputter-coated with gold. Imaging was performed using a Zeiss Sigma 300 FEG-SEM at an accelerating voltage of 2 kV.

X-ray diffraction (XRD) diffractograms were obtained using a Bruker D8 Advance Eco powder diffractometer with Cu Kα1,2 radiation. Patterns were collected over a 2θ range of 15–60° with a step size of 0.031°.

### Loading Quantification

Irinotecan loading was quantified by UV–Vis spectroscopy using a Shimadzu UV-1800 spectrophotometer, employing both indirect measurements of the supernatant after synthesis and direct measurements following particle dissolution in acid. Beer–Lambert calibrations based on absorbance at 220 nm were conducted to relate absorbance to irinotecan concentration for both protonated Irinotecan (as the hydrochloride salt, Irinotecan HCl) and unprotonated Irinotecan (prepared from 1 mM Irinotecan HCl + 5 mM Na_2_CO_3_ prepared then diluted to relevant conditions).

UV–Vis spectra were recorded over the 200–400 nm range for both the synthesis supernatant (100x dilution) and the solution obtained after particle dissolution, prepared by adding 200 μL of 1 M HCl to a 1 mg mL^–1^ particle suspension followed by a 10x dilution, to quantify drug loading.

### Particle Dissolution in Deionized Water Experiments

Dissolution experiments were carried out using an inverted optical microscope to monitor the projected area of individual particles in real time, as described in Milner et al.^17,18^ Synthesized irinotecan loaded vaterite particles were dispersed in 10 mL deionized water (4 μg mL^–1^) and introduced into an observation chamber, suspended above the objective lens of the microscope. Once a portion of the particles settled on the chamber bottom (≈10 s), time-lapse imaging was initiated at 10 s intervals. Observations were made using a 20x objective lens (Olympus UPLXAPO 20x) with LED phase-contrast illumination (Aura Pro, Cairn Research, Kent, U.K.), and images were captured with an ORCA-Flash 4.0 digital camera (C13440–20CU, Hamamatsu Photonics, Japan), to provide 16-bit, 4-megapixel images.

To investigate the effect of Fe­(II) on the dissolution rate of pure vaterite particles, the same procedure was followed, with the particles suspended at 4 μg mL^–1^ in aqueous solutions of FeSO_4_.7H_2_O (≥99%, Fisher Scientific) at concentrations of 3, 20, and 80 μM.

### Drug Release UV–Vis Experiment

To monitor the time-dependent release of irinotecan from irinotecan-loaded vaterite particles, a large-volume suspension (60 mL) of particles at a concentration of 40 μg mL^–1^ in deionized water was prepared in a conical flask and stirred continuously using a magnetic stir bar. Immediately after stirring commenced, an aliquot of the suspension was transferred to a cuvette using a micropipette, and an absorption spectrum was acquired over the 200–400 nm range using a Shimadzu UV-1800 spectrophotometer. This sampling procedure was repeated every 2 min from 0 to 10 min, every 5 min from 10 to 30 min, and once more after a total stirring time of 40 min, with all samples taken from the same stirred suspension. The experiment was repeated using three independently synthesized batches of irinotecan-loaded vaterite particles, and the absorbance at 220 nm was averaged across replicates at each time point. The first data point is plotted at t = 0.5 min to account for the time required to acquire the initial measurement.

### Particle Dissolution in Human Serum

To study the dissolution of vaterite and irinotecan-loaded vaterite particles in human serum using the same inverted optical microscopy setup employed for dissolution in water, the experimental procedure was slightly modified. Initial experiments using 10 mL suspensions at a particle concentration of 4 μg mL^–1^ were hindered by the opacity and viscosity of human serum, which reduced image focus and caused increased particle sedimentation onto the observation chamber surface. Consequently, 5 mL suspensions at a reduced concentration of 1 μg mL^–1^ were used for dissolution studies in neat human serum and all human serum–deionized water mixtures. Notably, the dissolution rate measured in 5 mL of deionized water at 1 μg mL^–1^ was the same to that obtained in 10 mL of deionized water at 4 μg mL^–1^.

The dissolution of vaterite particles was investigated under 14 different conditions, comprising pure human serum (HS), pure deionized (DI) water, and a series of intermediate HS/DI water mixtures. Dissolution of irinotecan-loaded vaterite particles was examined under four conditions: pure HS, pure DI water, and two intermediate mixtures.

### Data Analysis

Optical microscopy images were analyzed using ImageJ (Fiji). Particle projected areas were obtained using the “Default” autothresholding algorithm and converted from pixel counts to physical areas using the calibrated pixel-to-distance ratio. ImageJ was also used for SEM image analysis. Data plotting and fitting for dissolution and XRD measurements were performed using OriginPro 2023.

### Cell Culture

Capan-1 was cultivated in RPMI medium (Thermo Fisher Scientific, Waltham, MA, USA) containing 20% FBS (Thermo Fisher Scientific), 4 mM l-Glutamine (Thermo Fisher Scientific) and 1% Penicillin/Streptomycin (Thermo Fisher Scientific). MiaPaca-2 was cultivated in DMEM medium (Thermo Fisher Scientific) containing 20% FBS, 4 mM l-Glutamine and 1% Penicillin/Streptomycin. Mycoplasma tests were performed after thawing using the Mycoplasma detection kit (Applied biological Materials, Richmond, Canada) to ensure that cells were mycoplasma-free. Medium was exchanged every two to 3 days and cells were passaged when reaching 80% confluency. For passaging, cells were incubated with Trypsin/EDTA for 5 min. The detachment was stopped by adding medium containing FBS. The cell suspension was centrifuged for 5 min at 1200 rpm After centrifugation, cells were resuspended in cell culture medium and plated at a dilution of 1:5.

### Pharmacotyping

For drug testing, cells were stained with Acridine Orange/Propium Iodide stain (BioCat, Heidelberg, Germany) and counted using the LUNA automated cell counter (BioCat, Heidelberg, Germany). 3000 cells were seeded per well in a 96-well plate. Twenty-4 h after seeding, drugs were added in triplicates in a dilution series ranging from 13 nM to 50 μM. Irinotecan was dissolved in DMSO, irinotecan-loaded vaterite was dissolved in NaCl to a stock concentration of 25 mM. After 4 days of incubation, the CellTiter-Glo Luminescent Cell Viability Assay (Promega, Walldorf, Germany) was performed to measure cell viability. Luminescence measurements were conducted using the VICTOR Nivo multiplate reader (Revvity Inc., Waltham, MA, USA). Dose–response curves were calculated in GraphPad Prism (Version 10.6.0).

## Results and Discussion

Either a coprecipitation or an adsorption route may be employed for loading biomolecules into porous vaterite. In the case of coprecipitation, high loading can be achieved with water-soluble drugs through simultaneous vaterite formation and drug incorporation. In contrast, an adsorption route is preferred for hydrophobic drugs that can be dissolved in organic solvents and mixed with preformed vaterite particles. Given its good water solubility, irinotecan hydrochloride was incorporated via a coprecipitation route. The loading of irinotecan into the particles was estimated using UV–Vis spectroscopy, both indirectly from the supernatant after synthesis and directly by dissolving the particles after isolation showing loadings in excess of 100 μmol/g. Further discussion of the method and data used to estimate loading is provided in Section 1 of the Supporting Information.

To further characterize the irinotecan-loaded particles, XRD and SEM analyses were performed. The XRD diffractogram (Section 2, Supporting Information) confirms vaterite to be the overwhelmingly dominant calcium carbonate polymorph. Figure 1 presents an SEM image of the irinotecan loaded particles and of a vaterite particle prepared using identical synthesis but without irinotecan. Spherical microparticles composed of nanosized, approximately spherical subunits, characteristic of vaterite, are clearly visible in both cases. In the case of irinotecan-loaded vaterite, the samples exhibited traces of nonvaterite material on the surface of the vaterite particles.

**Figure 1:**
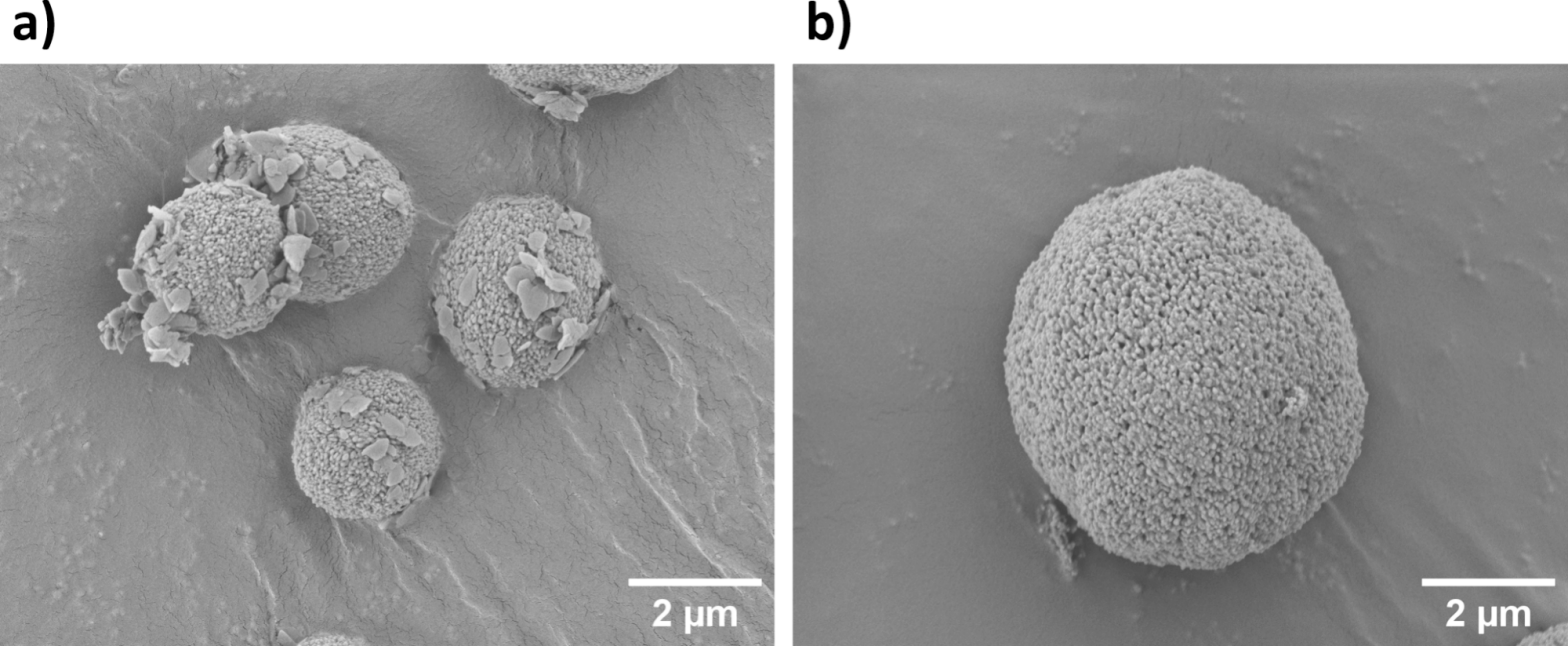
SEM images of (a) irinotecan-loaded vaterite and (b) vaterite at 10K× magnification.

The average diameter of nonagglomerated particles is 3.0 ± 0.7 μm, compared with 3.7 ± 0.6 μm for vaterite particles prepared without irinotecan. This indicates the presence of the drug does not perceptibly alter polymorph formation or particle growth. The nonvaterite surface material may correspond to irinotecan and is discussed further below. Further discussion and images are provided in the Supporting Information, Section 2, along with a report of the partial particle aggregation, which is relevant to the discussion that follows.

The dissolution behavior of irinotecan-loaded vaterite particles was investigated using optical microscopy.^17,18^ Based on prior work,^17–19^ this approach allows the projected area of individual particles to be measured and plotted as a function of time as they dissolve under controlled conditions with a well-defined diffusive mass-transport regime. Within the established model, this approach enables distinction between thermodynamic control and kinetic control. In the thermodynamic controlled case, the dissolution rate is fixed by the solubility product and the diffusion of the dissolved solid, whereas for kinetic control the rate is determined entirely by interfacial reaction kinetics. For the specific case of calcium carbonate dissolution, these limiting regimes are illustrated by eqs 1 and 2 for thermodynamic and kinetic control, respectively:

$${CaCO}_{3}\left(s\right)\overset{k_{f}}{\underset{k_{b}}{⇌}}{Ca}^{2+}\left(aq\right)+{{CO}_{3}}^{2-}\left(aq\right)\overset{k_{mt}}{\to }\mathrm{bulk}$$ 1

$${CaCO}_{3}\left(s\right)\overset{k_{ir}}{\to }{Ca}^{2+}\left(aq\right)+{{CO}_{3}}^{2-}\left(aq\right)$$ 2

Rate constant *k*_f_ corresponds to dissolution, and *k*_b_ to precipitation; *k*_mt_ is the mass transport rate constant, and *k*_ir_ the rate constant for dissolution when only interfacial kinetics are important. Considering these regimes and the data extracted from the optical microscopy setup, thermodynamic control can be identified by a linear change in projected area with time, whereas kinetic control is characterized by a linear variation in diameter with time. For pure vaterite monomer particles, thermodynamic control was observed, as evidenced by a linear change in particle projected area with time, with an average dissolution rate of (1.1 ± 0.1) × 10^–13^ m^2^ s^–1^.^17,18^

For the data set of irinotecan-loaded vaterite particles, the projected area decreases linearly with time whereas the diameter shows a nonlinear variation (Figure 2), consistent with dissolution under thermodynamic control rather than kinetic control. Linear fitting of the region corresponding to the first 50% reduction in area yielded an average dissolution rate of (1.1 ± 0.1) × 10^–13^ m^2^ s^–1^, effectively identical to the value obtained for pure vaterite.^17,18^ This allows the inference that the incorporation of irinotecan into vaterite does not affect the dissolution mechanism or kinetics of the particles, either qualitatively or quantitatively.

**Figure 2:**
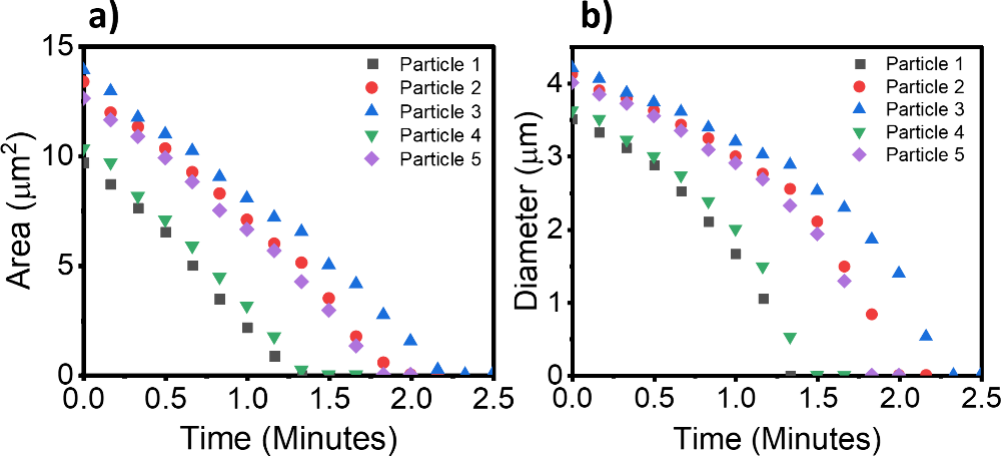
Measured (a) projected area and (b) diameter of five individual irinotecan-loaded vaterite particles as a function of time. The particles were suspended in deionized water at a concentration of 4 μg mL^–1^ and imaged using an inverted optical microscopy setup.

The time scale of irinotecan release was monitored by UV–vis spectroscopy. The absorbance at a wavelength corresponding to irinotecan concentration was averaged across the repeats and plotted as a function of time (Figure 3). An appreciable absorbance is observed in the initial measurement, followed by a gradual increase that reaches a steady value after approximately 15 min. The initially high absorbance is attributed to the release of irinotecan from the exterior surface and near immediate interior surface of the particles. Subsequently, the drug is released as particle dissolution proceeds, exposing fresh interior surfaces on which the drug is adsorbed. The subsequent gradual increase in absorbance over time is then observed as the particles continue to dissolve and release irinotecan into solution.

**Figure 3:**
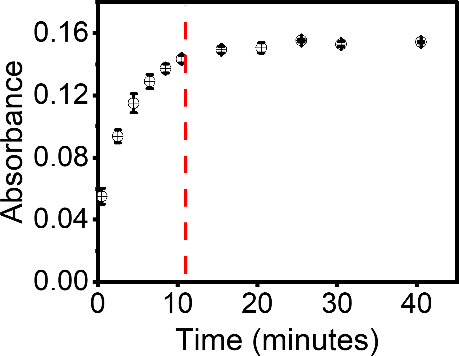
Time-dependent release of irinotecan from vaterite particles, monitored by absorbance. Samples were taken from a stirring 40 μg mL^–1^ suspension of irinotecan-loaded vaterite particles. Error bars represent the mean of three experiments; the dashed line indicates the average dissolution time inferred from optical microscopy.

To compare the time scale of irinotecan release with vaterite dissolution, the optical microscopy experiment was repeated at a particle concentration of 40 μg mL^–1^, revealing an average dissolution time of 11 ± 1 min for complete dissolution of a set of individual (nonaggregated) irinotecan-loaded particles. Plots of area versus time for the particles analyzed are included in Section 3 of the Supporting Information. As one can clearly see through visual inspection of Figure 3, the time scale of irinotecan release can be seen to correlate with particle dissolution. To extend this comparison, the time taken to reach different extents of either irinotecan release or particle dissolution was calculated and discussed in Section 4 of the Supporting Information. Again, the two are clearly correlated, but with irinotecan release occurring slightly faster than particle dissolution. This short time lag may result from the diffusion of some irinotecan out of the pores before the particles have fully dissolved.

To quantify the kinetics in a drug delivery context, the dissolution of both unloaded and irinotecan-loaded vaterite in human serum, both diluted and undiluted, was investigated using an inverted optical microscope. Adjustments were required due to the opacity and viscosity of human serum; see the discussion in Section 5 of the Supporting Information. The dissolution of vaterite particles was studied under 14 different conditions, including pure human serum (HS), pure deionized (DI) water, and a range of intermediate mixtures. The dissolution of irinotecan-loaded vaterite was studied under four conditions: pure HS, pure DI water, and two intermediate mixtures. Notably, the variation of projected area with time exhibited good linearity, indicating dissolution occurs under thermodynamic control regardless of the concertation of human serum; plots are provided in Section 5 of the Supporting Information. The average dissolution rate was extracted by linear fitting of the region corresponding to the first 50% reduction in projected area, using at least five particles for each condition. These values are plotted for vaterite and irinotecan-loaded vaterite as a function of human serum percentage (%HS, calculated from the volumetric fraction of human serum in deionized water) in Figure 4. For the vaterite particles, as the conditions are varied from 0% HS (pure deionized water) to 8.3% HS, the average dissolution rate decreases sharply by a factor of approximately 3. The dissolution rate then remains relatively constant and independent of the concentration of human serum up to 75% HS, after which it decreases further toward 100% HS (neat human serum). Overall, the dissolution rate decreases by approximately a factor of 5, from (9.6 ± 0.5) × 10^–14^ m^2^ s^–1^ in pure water to (2.1 ± 0.4) × 10^–14^ m^2^ s^–1^ in neat human serum. This trend is closely mirrored by the data collected for the irinotecan-loaded vaterite particles (Figure 4).

**Figure 4:**
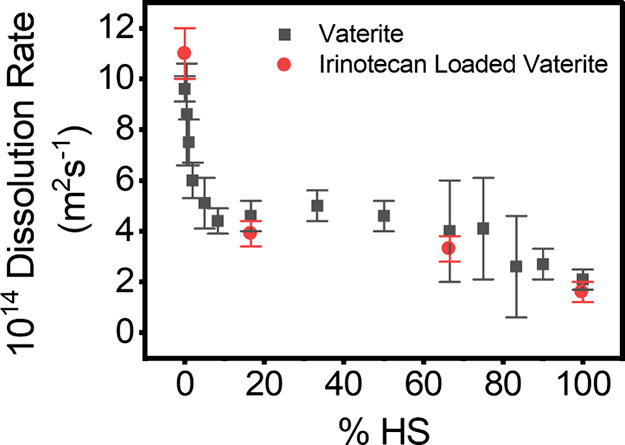
Plot of the average dissolution rate as a function of percentage human serum (%HS calculated as (volume of HS/total solution volume) × 100, based on dilution with deionized water) for vaterite particles (black) and irinotecan-loaded vaterite particles (red). Each data point corresponds to the average dissolution rate of at least five particles.

The two-stage concentration dependence of the dissolution rate suggests that viscosity effects alone are unlikely to account for the observed behavior; human serum has a relative viscosity of up to 1.6 compared to water.^28^ Instead, the formation of a protein corona, in which nano- and microparticles become enveloped by layers of proteins and biomolecules upon exposure to biological fluids, provides a more likely, albeit speculative, explanation.^29^ The sharp initial decrease in dissolution rate might reflect soft corona formation via rapid adsorption of abundant, low-affinity (i.e., weakly binding) proteins, followed at higher concentrations by hard corona formation from high-affinity (i.e., strongly binding) proteins that further inhibit dissolution. Note that the possible adsorption of Fe^2+^ was investigated as a potential rate inhibitor, motivated by its high abundance (total concentration, including bound ions, ∼9 mM) in the human serum metabolome and its potential for strong adsorption onto vaterite.^30^ However, this possibility was ruled out based on experiments outlined and discussed in Section 6 of the Supporting Information.

The antitumoral effect of irinotecan-vaterite was confirmed by performing in vitro drug tests on two established pancreatic cancer cell lines, MiaPaca-2 and Capan-1. Irinotecan and irinotecan-vaterite were added in 10 concentrations ranging from 13 nM to 50 μM, as previously described by Beutel et al.,^31^ and cell viability was measured after incubation for 4 days. Figure 5 shows the corresponding dose–response curves. The IC_50_, indicating the drug concentration at which the cell viability was reduced by 50%, was used to quantify the antitumoral activity of both drugs. Irinotecan resulted in a reduction in cell viability at concentrations above 0.5 μM with IC_50_ values of 24.18 μM for MiaPaca-2 and 3.49 μM for Capan-1 cells. These IC_50_ values are comparable to previously published results.^32–34^ Higher concentrations of irinotecan-vaterite were required for equivalent cell viability reductions. The IC_50_ values were 113.90 μM for MiaPaca-2 and 29.89 μM for Capan-1. This might be explained by a reduced effect of the irinotecan-vaterite as not all irinotecan might have been liberated into the cell culture medium at physiological pH.

**Figure 5:**
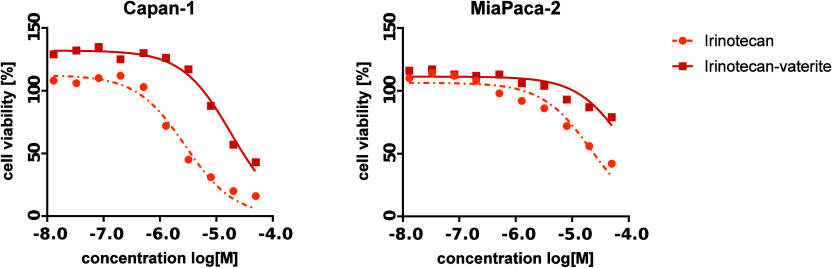
Dose–response curves of irinotecan and irinotecan-vaterite in Capan-1 and MiaPaca-2 cells. Normalized cell viability is plotted as a function of concentration of the corresponding drug.

## Conclusions

Beyond the synthesis of novel irinotecan-loaded vaterite particles, this work demonstrates a clear correlation between particle dissolution and drug release. The experimental approach used here can be readily applied to other systems in which either the drug or the carrier has been varied. Dissolution studies in water provide fundamental mechanistic and kinetic insight into the behavior of the system, while extension of these studies to human serum offers comparable insight under conditions more relevant to in vivo application. Dissolution experiments in human serum/water mixtures reveal a pronounced concentration dependence, with the dissolution rate decreasing in two distinct regimes, which is tentatively attributed to protein corona formation. Finally, the irinotecan-loaded vaterite particles are shown to exert a therapeutic effect in two human pancreatic cancer cell lines. These results are promising for future clinical applications. By enabling targeted release at the acidic tumor site, irinotecan-loaded vaterite can potentially reduce adverse side effects while increasing its concentrations and antitumoral activity. Since irinotecan was found to be more toxic in in vitro experiments than irinotecan-loaded vaterite, one might speculate that not all irinotecan might have been liberated in the in vitro experiments that were conducted at physiological pH. Thus, more extensive in vitro studies at various different pH ranges will be in the future to assess the full extent of the delivery of vaterite in biological systems. This work could accompany kinetic studies of drug-loaded systems in varied pH biological mimicking environments; this is complicated by the buffering capacity of systems such as human serum. Nevertheless, our results demonstrate the feasibility of irinotecan-vaterite as a drug delivery system, thus supporting its clinical implementation in the future.

## Supplementary Material



## Notes

UV–vis spectra and analysis for particle loading quantification, XRD diffractograms and SEM images with extended size analysis of irinotecan-loaded vaterite, representative raw images and dissolution data in deionized water, expanded analysis of irinotecan release and its correlation with dissolution behavior, raw data and area–time plots for vaterite and irinotecan-loaded vaterite dissolution in human serum, and additional data and discussion on the effect of Fe­(II) on vaterite dissolution (PDF)
