# Supplementary material for: Irinotecan-Loaded Vaterite Microspheres for Drug Delivery: Drug Release and Dissolution Kinetics and Mechanism in an Aqueous Solution and Human Serum
Source: Langmuir. 2026 May 27;42(22):15658–64. doi: 10.1021/acs.langmuir.6c01242 (PMC13262042; doi:10.1021/acs.langmuir.6c01242)
Supplement: Supplementary file 1 [file la6c01242_si_001.pdf]

Supporting Information - Irinotecan Loaded Vaterite Microspheres for Drug Delivery: Drug Release and Dissolution Kinetics and Mechanism in Aqueous Solution and Human Serum

Authors: *Morgan P. Milner [a], Hugo A. Saint [b], Jake M. Yang [c], Christopher C.M. Neumann[d], Katharina Wansch[d], Richard G. Compton[*a]*

a) Physical and Theoretical Chemistry Laboratory, Department of Chemistry, University of Oxford, Oxford OX1 3QZ, Great Britain

b) St John's College, St Giles, Oxford OX1 3JP, Great Britain

c) Centre for Sustainable Materials Processing, School of Chemistry, University of Leicester, Leicester LE1 7RH, Great Britain

d) Department of Hematology, Oncology and Tumor Immunology, Charité-Universitätsmedizin Berlin, Freie Universität Berlin, Humboldt-Universität zu Berlin, Berlin Institute of Health, Charitéplatz 1, D-10117 Berlin, Germany

Table of Contents

Section 1. Irinotecan Loaded Vaterite Synthesis and Loading Quantification……………………S3

Section 2. Particle Characterization..S7

Section 3. Particle Dissolution Kinetics in Deionized Water..S10

Section 4. Irinotecan Release and Correlation with Particle Dissolution....................................S12

Section 5. Particle Dissolution in Human Serum..S15

Section 6. Effect of Fe(II) on the Dissolution of Vaterite.. S26

References…..S29

Section 1. Irinotecan Loaded Vaterite Synthesis and Loading Quantification

Irinotecan loaded vaterite particles were prepared by a simple co-precipitation method outlined in the Experimental Section of the main text.

Irinotecan loading was quantified using UV–Vis spectroscopy, both indirectly by measuring the amount remaining in the supernatant after synthesis and directly by determining the amount released after dissolving the particles in a small volume of acid. To correlate absorbance with irinotecan concentration, a Beer–Lambert calibration was employed. During synthesis, protonated irinotecan is deprotonated by carbonate ions, whilst upon particle dissolution it is protonated again by the added acid. UV–Vis spectra for a range of concentrations (5–50 μM) of both irinotecan HCl and deprotonated irinotecan (1 mM Irinotecan HCl + 5 mM Na_2CO_3 prepared then diluted to relevant conditions) are shown in Figure S1. While minor differences appear in the spectral region between 325 and 400 nm and the peak at around 255 nm, the peak at 220 nm is identical in form in both cases. The differences observed are consistent with prior publication on Irinotecan and its protonated form.[1] Accordingly, Beer–Lambert calibrations based on absorbance at 220 nm are presented in Figure S2, yielding molar extinction coefficients of $4.4 \pm 0.06 \times 10^4$ L mol^{-1} cm^{-1} and $4.2 \pm 0.07 \times 10^4$ L mol^{-1} cm^{-1} for the protonated and deprotonated forms, respectively.

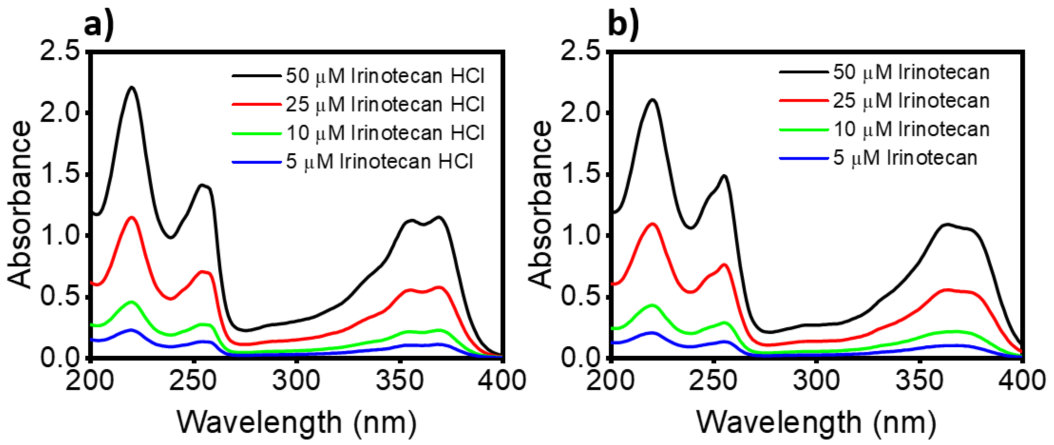

Figure S1. UV-VIS absorption spectra in the 200-400 nm range of a) Irinotecan HCl and b) Irinotecan (deprotonated by preparing 1 mM Irinotecan HCl + 5 mM Na_2CO_3 then diluting); spectra were recorded at 5 different concentrations for Beer-Lambert calibration.

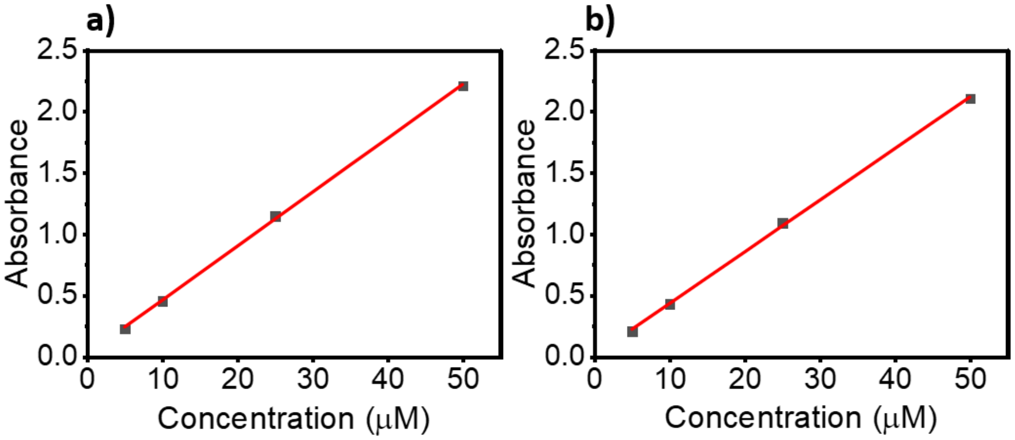

Figure S2. Absorbance at 220 nm plotted as a function of concentration of a) Irinotecan HCl and b) Irinotecan (deprotonated by preparing 1 mM Irinotecan HCl + 5 mM Na_2CO_3 then diluting). A linear fitting was applied in both cases to extract a molar extinction coefficient in accordance with the Beer-Lambert law.

To estimate drug loading both indirectly from the supernatant and directly from the particles, five independent synthesis were conducted and the particles analyzed. UV-Vis spectra of the

supernatant (100 x dilution) and of the solution obtained after dissolving a suspension of the particles (200 uL 1M HCl added to a 1mg/mL particle suspension then 10 x dilution prepared) were recorded in the 400 - 200 nm range. The raw data for this analysis is included in Figure S3. The absorbance at 220 nm was used to determine the irinotecan concentration, which was then converted to drug loading based on the total mass of vaterite produced for the supernatant-based estimate, or the mass of vaterite dissolved for the direct measurement. This yielded loading values of 91.5 ± 10 mg/g and 65.5 ± 2.3 mg/g in mass terms, or 156 ± 18 μmol/g and 112 ± 4.0 μmol/g in molar terms respectively.

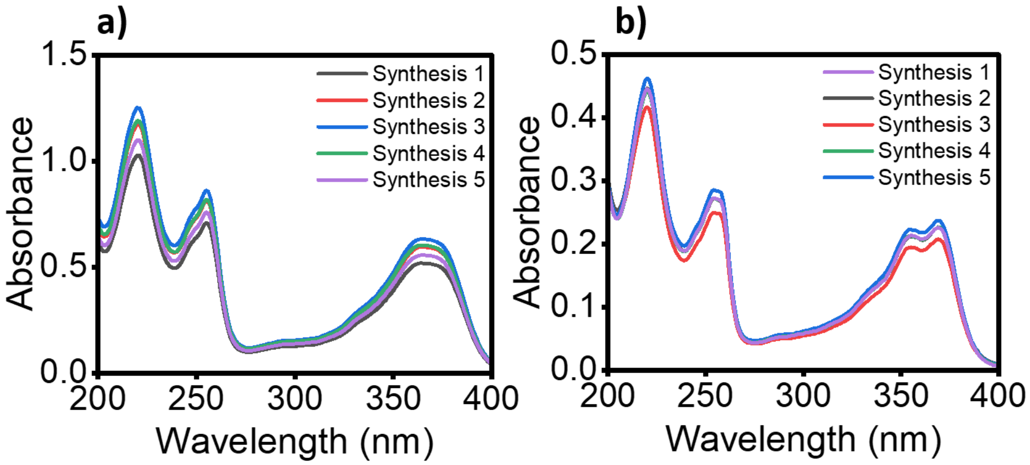

Figure S3. UV-VIS absorption spectra in the 200-400 nm range of a) the supernatant (100x dil.) and b) the resulting sample from diluting particles with acid, for 5 synthesis of irinotecan loaded particles.

The higher loading estimated from the supernatant compared with the direct measurement from dissolved particles is likely due to partial loss of irinotecan from the vaterite during the washing steps. Although indirect estimates from the supernatant are commonly reported, see for example, [2,3] direct measurement likely provides a more accurate and application-relevant assessment of drug

loading. Both values are comparable to those reported in the literature for vaterite loaded with other drugs (Table S1), which also summarizes the incorporation conditions. Drug loading in the literature is often reported as the mass of drug per mass of vaterite; however, in this work the drug mass has been converted to moles to enable better comparison. Notably, there are relatively few reported examples of small-molecule chemotherapeutics being incorporated into vaterite without the use of shells or additives, and accurate reporting of loading in such systems remains rare.

Paper	Particles	Load	Incorporation Quantification
https://doi.org/10.1088/2057-1976/aa9719	Vaterite nanoparticles	cisplatin	Loading = 178 µmol/g<br>LE= 83.56%
10.1016/j.msec.2012.08.026	Vaterite microparticles with BSA additive	camptothecin	Loading = 4.28 µmol/g<br>LE = 11.05%.
https://doi.org/10.1016/j.ijpharm.2019.118866	Vaterite nanoparticles	Curcumin	LE = 70%<br>Loading = 138 µmol/g
https://doi.org/10.1016/j.colsurfb.2014.09.047	Alginate/$CaCO_3$ hybrid nanoparticles	Paclitaxel + DOX	Loading = 110 µmol/g<br>LE = 46.9%<br>(for paclitaxel)
https://doi.org/10.1016/j.mtcomm.2024.109451	Vaterite micro and cub micron particles	mitoxantrone	Micron:<br>Loading = 202.5 µmol/g<br>LE = 70 ± 9 %<br>Sub-micron:<br>Loading = 157.5 µmol/g<br>LE = 65 ± 14 %

Table S1. Literature summary of chemotherapeutic drugs incorporated into vaterite, for literature that reports loading without incorporating a shell formation strategy.[2–6]

Section 2. Particle Characterization

X-ray diffraction was used to confirm the formation of the vaterite phase of calcium carbonate and to demonstrate that the presence of irinotecan does not significantly affect the synthesis, for example and in particular by promoting the formation of other polymorphs. The diffractogram for the irinotecan-loaded particles is shown in Figure S4, and all prominent peaks are assigned to vaterite in accordance with literature.[7] The broad peak observed over the approximate range of 2θ = 18–38° is associated with amorphous calcium carbonate and is clearly visible, unlike in the diffractogram of the equivalent vaterite synthesis without irinotecan.[8,9] However, all sharp peaks correspond to vaterite, in accordance with the literature, and remain clearly dominant.

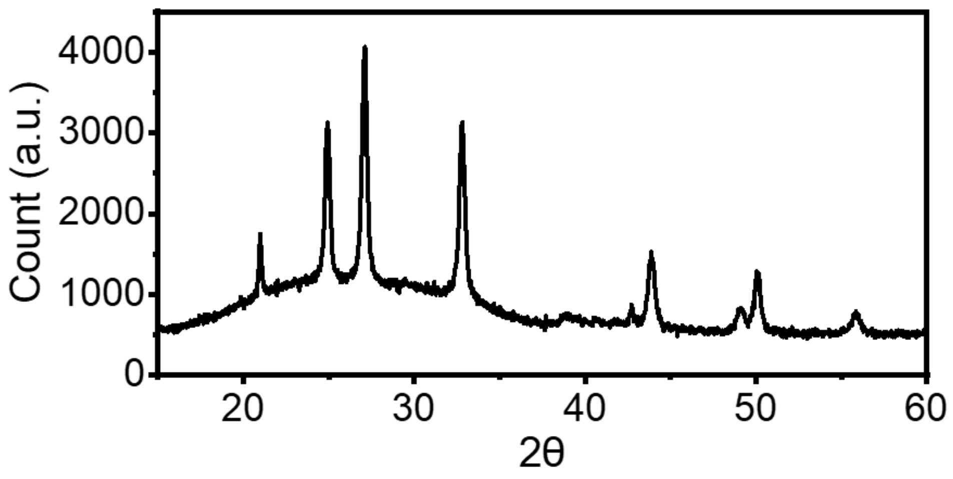

Figure S4. XRD diffractogram of prepared Irinotecan loaded vaterite particles, all prominent peaks are characteristic of the vaterite polymorph of calcium carbonate.[7]

SEM images of both irinotecan-loaded vaterite and unloaded vaterite particles were obtained at high (10k x) and low (1k x) magnification to examine surface morphology and to assess overall particle size and aggregation, respectively. Representative high-magnification images are shown

in the main text (Figure 1), while Figure S5 presents low-magnification images used to quantify average particle size and the extent of aggregation.

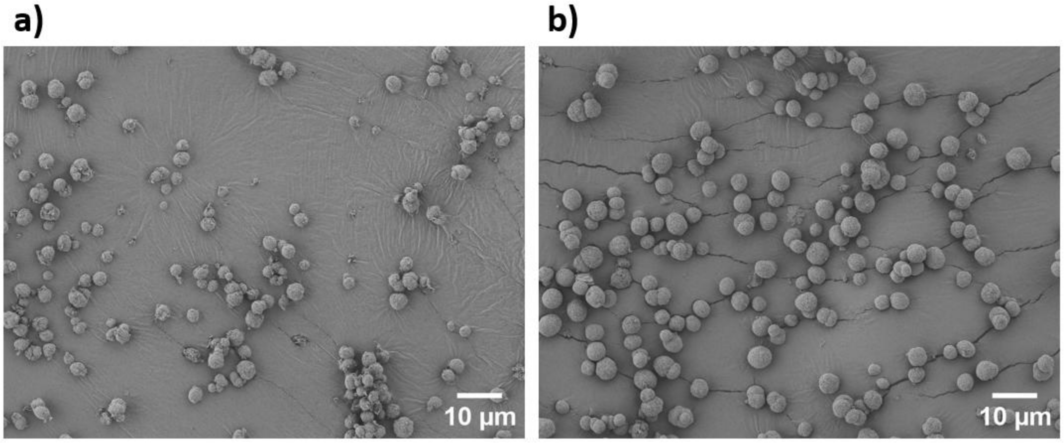

Figure S5. SEM images of a) irinotecan loaded vaterite and b) vaterite at 1k x magnification.

Average particle size was determined by manually outlining the projected area of 150 irinotecan-loaded vaterite or unloaded vaterite particles for each dataset. These areas were converted to diameters assuming spherical geometry. The mean diameter of non-agglomerated irinotecan-loaded particles was found to be 3.0 ± 0.7 μm, compared with 3.7 ± 0.6 μm for vaterite particles prepared without irinotecan, corresponding to areas of 7.2 ± 3 μm^2 and 11 ± 4 μm^2, respectively. Particle size distributions are shown in Figures S6 and S7.

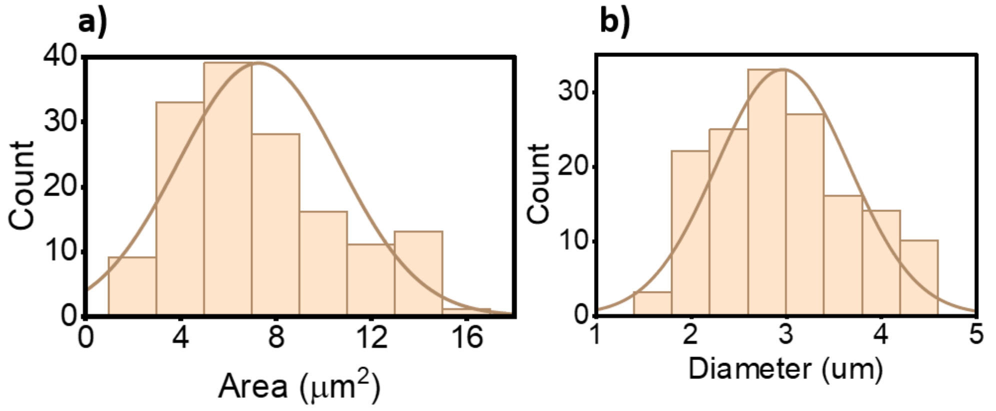

Figure S6. Particle a) area and b) diameter size distributions for irinotecan loaded vaterite particles.

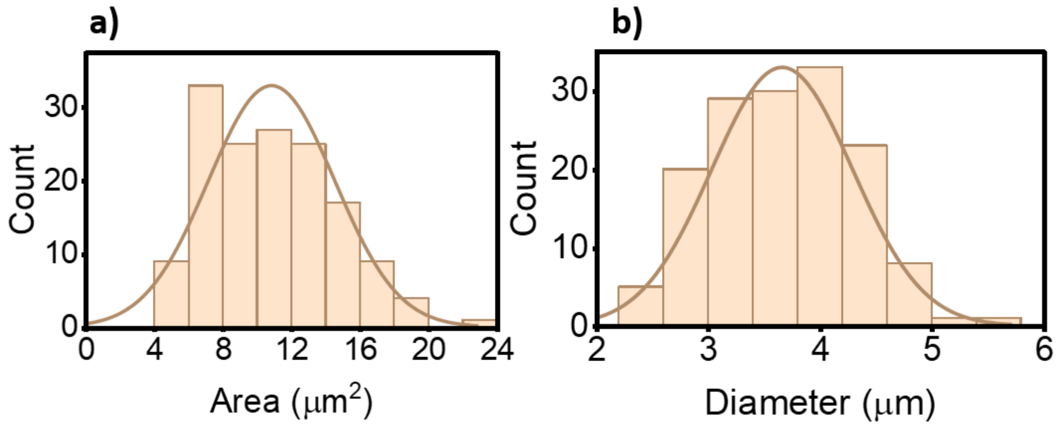

Figure S7. Particle a) area and b) diameter size distributions for vaterite particles.

To assess the extent of aggregation, low-magnification SEM images were used to classify particles visually as either non-aggregated monomeric particles or aggregated particles composed of irreversibly attached monomers. For each dataset, ~ 150 particles were analyzed and particles belonging to these categories were assigned. For the irinotecan-loaded vaterite samples, the monomer-to-aggregate ratio is approximately 2:1, whereas for vaterite particles synthesized without irinotecan, it is close to 3:1. Although the extent of aggregation is evidently higher in the irinotecan-loaded vaterite, monomers remain clearly dominant.

Section 3. Particle Dissolution Kinetics in Deionized Water

A dissolution study was conducted using an inverted optical microscopy setup, based on prior studies, for irinotecan-loaded vaterite at the standard particle concentration of 4 μg mL^{-1} to minimize diffusional overlap.[8,9] Particles were suspended in deionized water and added to an observation chamber. Image capture was initiated once some particles had settled on the surface and focus had been optimized, and images were then acquired at regular time intervals. The projected area of the particles was extracted using an auto-thresholding algorithm; an example of the extracted data before and after applying this algorithm, for a representative particle in this data set, is given in Figure S8. Plots of particle area versus time for the studied samples are shown in Figure 4 of the main text. A clear linear variation of area with time was observed, and linear fitting yielded an average dissolution rate of $1.1 \pm 0.1 \times 10^{-13}$ m^2s^{-1} the same as reported for pure vaterite.[8,9]

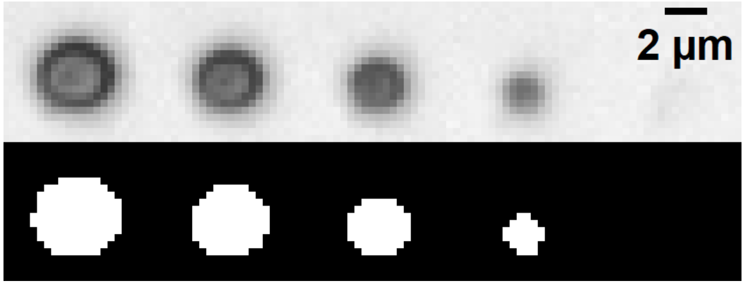

Figure S8. A series of optical microscopy images showing the dissolution of an irinotecan loaded vaterite particle in deionized water at a particle concentration of 4 μg mL^{-1}. Images before (top) and after (bottom) application of the auto-thresholding algorithm are shown. The extracted data correspond to Particle 5 in the size against time plots (Figure 2) of the manuscript.

Dissolution experiments were also performed at a higher particle concentration of 40 μg mL^{-1} to compare the dissolution timescale with that of the irinotecan release study conducted using UV spectroscopy, as presented in the main text. Plots of area versus time for the particles at this concentration are shown in Figure S9. At 40 μg mL^{-1}, the variation of area with time is less clearly linear; however, for the selected vaterite monomers, dissolution is complete within an average of 11 ± 1 minutes.

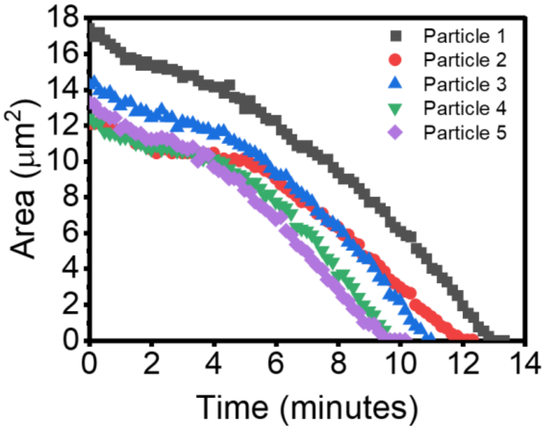

Figure S9. Particle area time plots for Irinotecan loaded vaterite particles at a concentration of 40 μg mL^{-1} in deionized water.

Section 4. Irinotecan Release and Correlation with Particle Dissolution

To investigate the time-dependent release of irinotecan from vaterite particles, UV–Vis measurements were performed on a stirred suspension of irinotecan-loaded vaterite particles at a concentration of 40 μg mL^{-1}, with samples taken at defined time intervals. Representative UV–Vis spectra are shown in Figure S10. A decreasing absorbance at 400 nm is observed over time, in contrast to the near-zero absorbance at this wavelength in the Beer–Lambert calibration (Figure S1). This signal is attributed to light scattering from the vaterite particles, which diminishes as the particles dissolve. To obtain a more accurate measure of irinotecan release, baseline correction was applied by setting the absorbance at 400 nm to zero, after which the corrected absorbance at 220 nm was used for analysis. The absorbance at 220 nm, previously correlated with irinotecan concentration, is presented as a function of time for three independently synthesized particles in Figure S11, and the corresponding average from three independent experiments is plotted in Figure 4 of the main text. The resulting data show an initial release prior to the first measurement, followed by a gradual release of irinotecan over approximately 15 minutes.

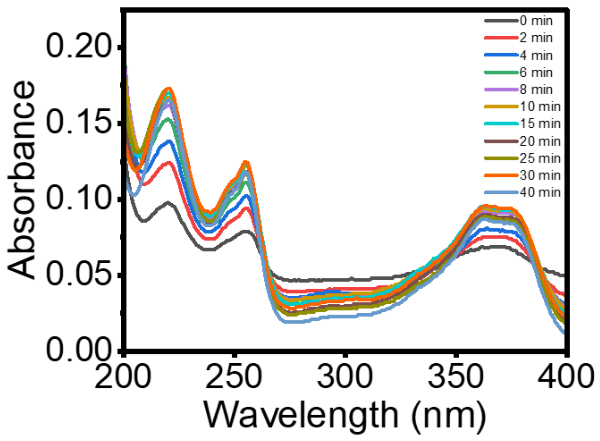

Figure S10. Example raw UV–Vis absorption spectra (Particles 1), recorded from samples taken at different time intervals from a stirred suspension of irinotecan-loaded vaterite particles at a concentration of 40 μg mL^{-1}.

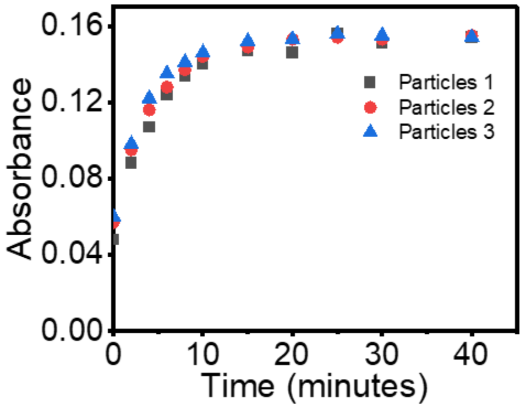

Figure S11. Release of irinotecan from three independently synthesized batches of irinotecan-loaded vaterite particles, presented as the increase in absorbance at 220 nm as a function of time. Baseline correction was applied to remove light-scattering contributions from the vaterite particles, ensuring that the absorbance at 220 nm accurately reflects irinotecan concentration. Samples were collected from a stirred suspension of irinotecan-loaded vaterite particles at a concentration of 40 μg mL^{-1}.

This was compared with vaterite dissolution data from the optical microscopy experiment to see if particle dissolution and irinotecan release are correlated. Table S2 presents the times taken for different extents of dissolution and drug release from optical microscopy or UV-Vis experiments respectively. For the UV/vis data the timescale for release subtracting the absorbance at t = 0.5 mins (the first measurement) is included, this allows better comparison as the surface irinotecan will be washed off before vaterite dissolution starts. The irinotecan release and particle dissolution can be seen to happen on a similar timescale. The slightly quicker irinotecan release than vaterite dissolution can be rationalised based on some irinotecan being able to diffuse out of the vaterite pores before the particles dissolve.

Comparison of the irinotecan release experiment and the dissolution experiment requires extrapolation from dissolution in 3D and with stirring, in the release experiment, to 2D and static, in the dissolution experiment. However, it is still noteworthy that the approximately 15 minutes required for the majority of Irinotecan to be released is similar to the time taken for single vaterite particle dissolution seen in these experiments (11± 1 minutes).

Extent of Irinotecan release	Average time take (40 ug/mL UV-vis study – relative to zero absorbance)	Average time take (40 ug/mL UV-vis study – relative to absorbance at first measurement)	Extent of dissolution	Average time take (40 ug/mL dissolution study)
25 %	~ 0 - 0.5 mins	~ 2 mins	25 %	4.9 ± 0.6
50 %	~ 2 mins	~ 3 mins	50 %	7.5 ± 0.9
75 %	~ 4.5 mins	~ 6.5 mins	75 %	9.3 ± 1

Table S2. Timescale for extent of dissolution/ drug release from optical microscopy or UV/vis experiments respectively.

Section 5. Particle Dissolution in Human Serum

The dissolution of vaterite and irinotecan-loaded vaterite particles in human serum was investigated using a slightly modified optical microscopy method, as described in the Experimental Section. Vaterite dissolution was examined under 14 conditions, including pure human serum (HS), pure deionized (DI) water, and a range of intermediate HS/DI mixtures. Irinotecan-loaded vaterite dissolution was studied under four conditions: pure HS, pure DI water, and two intermediate mixtures. For each condition, at least five particles were analyzed, and their projected area and equivalent diameter were plotted as functions of time. The projected area decreased linearly with time, whereas the diameter did not, indicating thermodynamic control of the dissolution process. A single particles area and diameter against time plot, that is representative of the data set, for each condition is presented in Figures S12 – S29. % human serum was calculated as (volume of HS / total solution volume) x 100, based on dilution with deionized water. Dissolution rates were therefore determined by linear fitting of the projected area–time data over the first 50% reduction in particle area and averaged for each condition, as presented in Figure 4 of the main text.

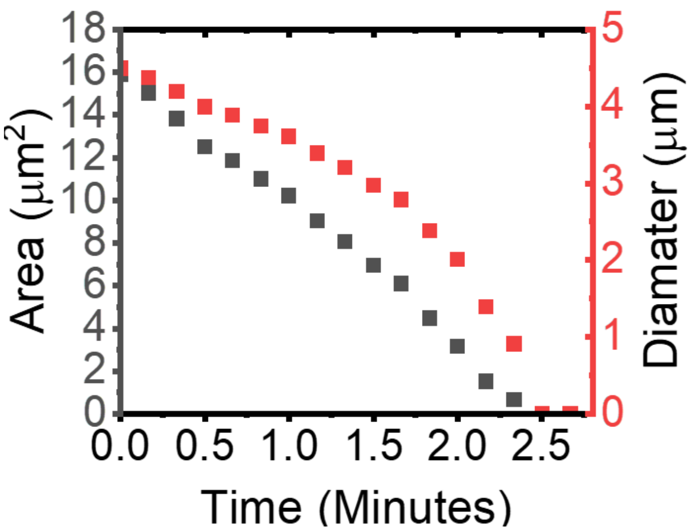

Figure S12. Measured projection area (black) and diameter (red) of a vaterite particle in 0 % human serum (pure water), plotted as a function of time at a particle concentration of 1 μg mL^{-1}.

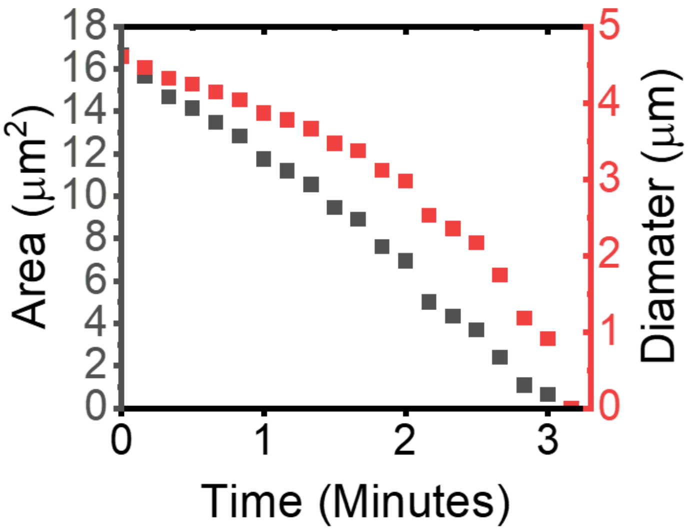

Figure S13. Measured projection area (black) and diameter (red) of a vaterite particle in 0.5 % human serum (calculated from the volumetric fraction of human serum in deionized water), plotted as a function of time at a particle concentration of 1 μg mL^{-1}.

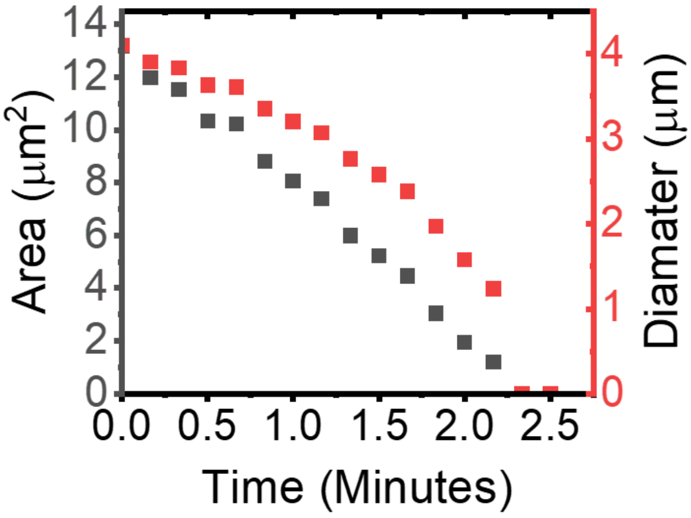

Figure S14. Measured projection area (black) and diameter (red) of a vaterite particle in 1 % human serum (calculated from the volumetric fraction of human serum in deionized water), plotted as a function of time at a particle concentration of 1 μg mL^{-1}.

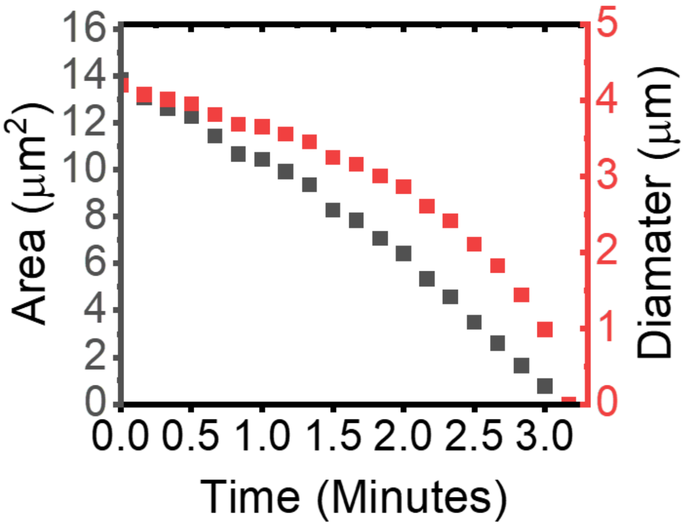

Figure S15. Measured projection area (black) and diameter (red) of a vaterite particle in 2 % human serum (calculated from the volumetric fraction of human serum in deionized water), plotted as a function of time at a particle concentration of 1 μg mL^{-1}.

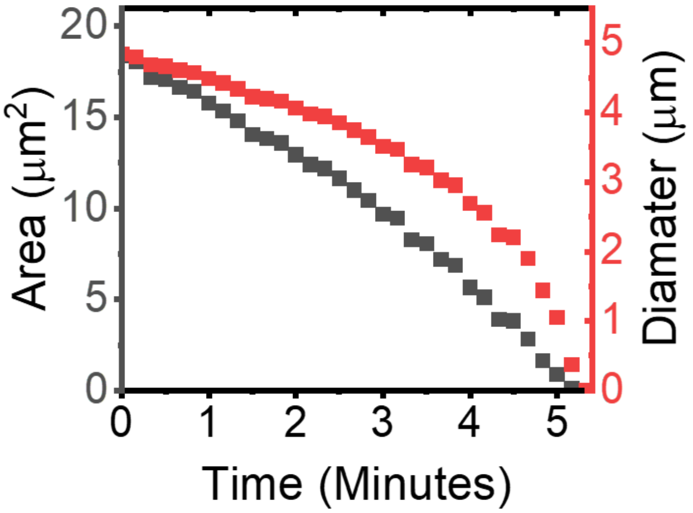

Figure S16. Measured projection area (black) and diameter (red) of a vaterite particle in 5 % human serum (calculated from the volumetric fraction of human serum in deionized water), plotted as a function of time at a particle concentration of 1 μg mL^{-1}.

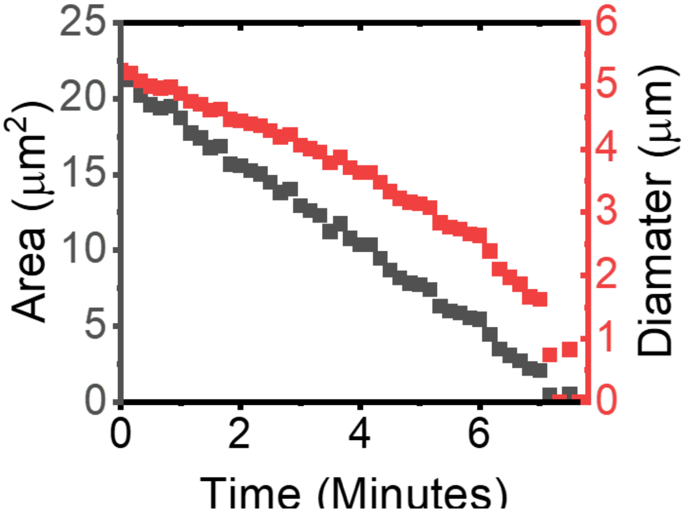

Figure S17. Measured projection area (black) and diameter (red) of a vaterite particle in 8.3 % human serum (calculated from the volumetric fraction of human serum in deionized water), plotted as a function of time at a particle concentration of 1 μg mL^{-1}.

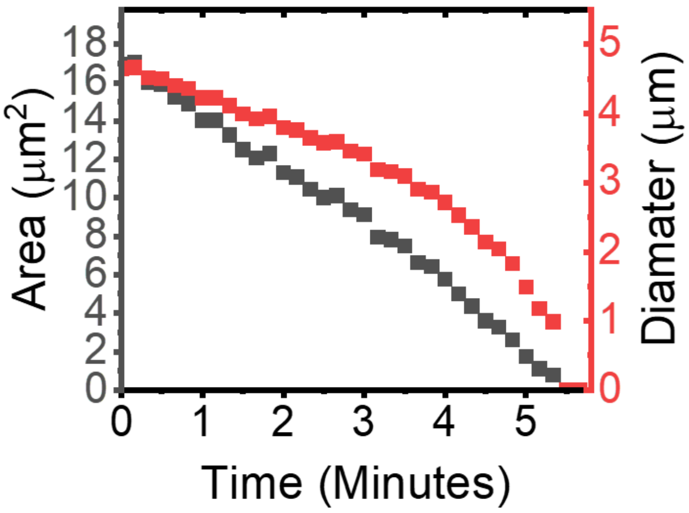

Figure S18. Measured projection area (black) and diameter (red) of a vaterite particle in 16.6 % human serum (calculated from the volumetric fraction of human serum in deionized water), plotted as a function of time at a particle concentration of 1 μg mL^{-1}.

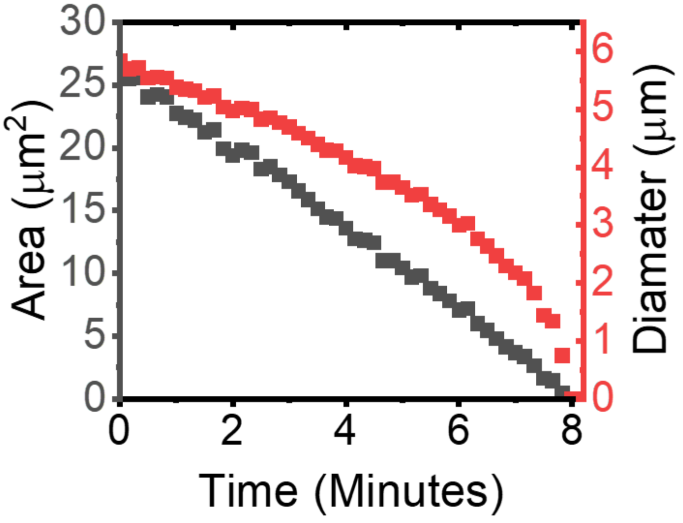

Figure S19. Measured projection area (black) and diameter (red) of a vaterite particle in 33.3 % human serum (calculated from the volumetric fraction of human serum in deionized water), plotted as a function of time at a particle concentration of 1 μg mL^{-1}.

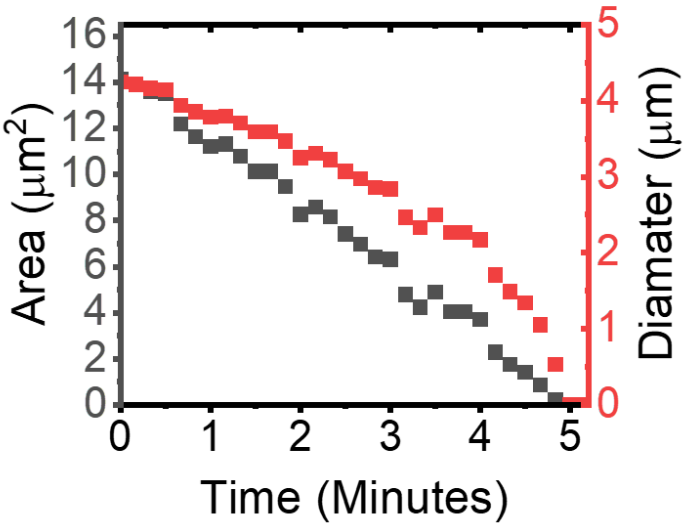

Figure S20. Measured projection area (black) and diameter (red) of a vaterite particle in 50 % human serum (calculated from the volumetric fraction of human serum in deionized water), plotted as a function of time at a particle concentration of 1 μg mL^{-1}.

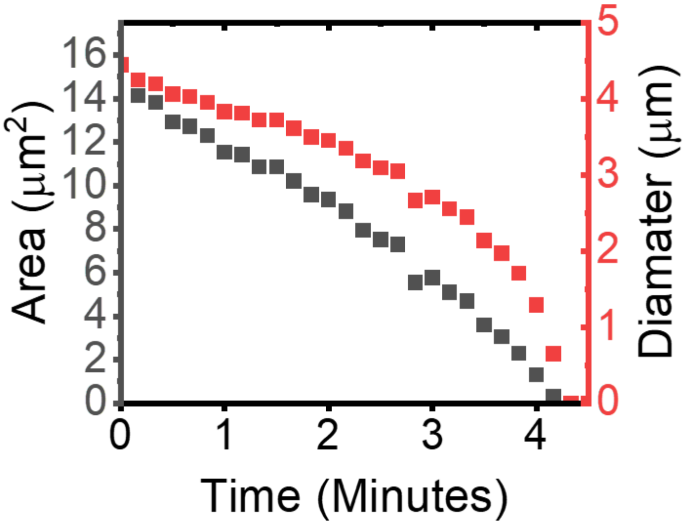

Figure S21. Measured projection area (black) and diameter (red) of a vaterite particle in 66.6 % human serum (calculated from the volumetric fraction of human serum in deionized water), plotted as a function of time at a particle concentration of 1 μg mL^{-1}.

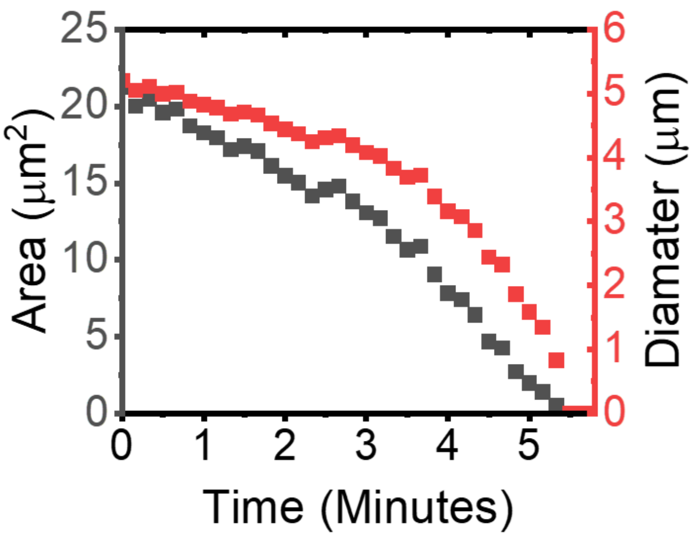

Figure S22. Measured projection area (black) and diameter (red) of a vaterite particle in 75 % human serum (calculated from the volumetric fraction of human serum in deionized water), plotted as a function of time at a particle concentration of 1 μg mL^{-1}.

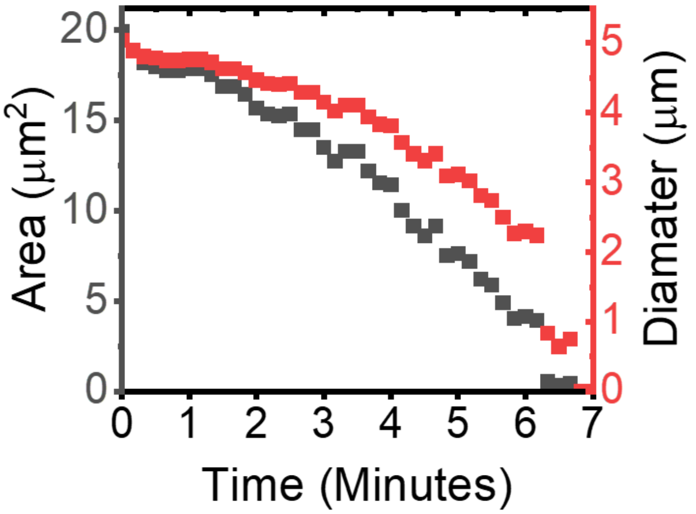

Figure S23. Measured projection area (black) and diameter (red) of a vaterite particle in 83.3 % human serum (calculated from the volumetric fraction of human serum in deionized water), plotted as a function of time at a particle concentration of 1 μg mL^{-1}.

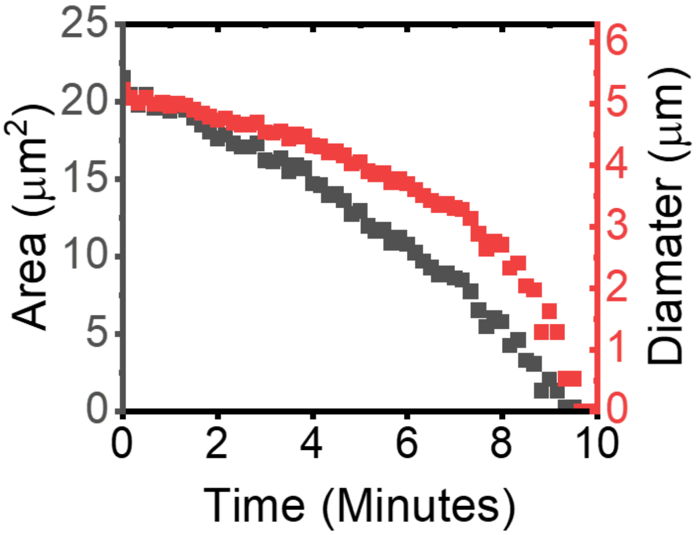

Figure S24. Measured projection area (black) and diameter (red) of a vaterite particle in 90 % human serum (calculated from the volumetric fraction of human serum in deionized water), plotted as a function of time at a particle concentration of 1 μg mL^{-1}.

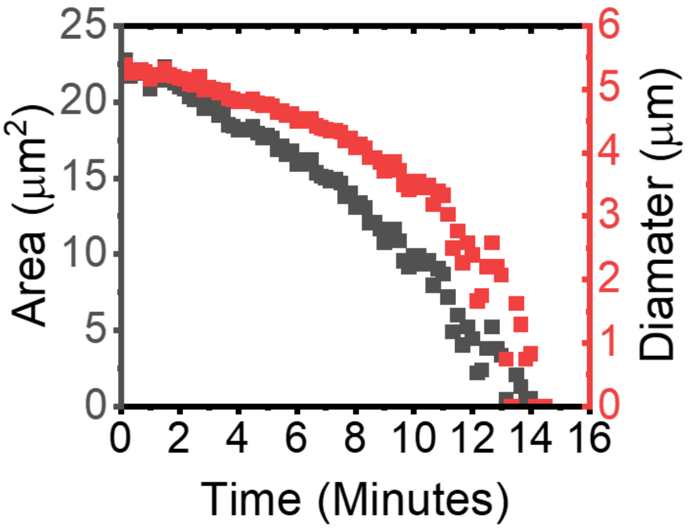

Figure S25. Measured projection area (black) and diameter (red) of a vaterite particle in 100 % human serum (pure), plotted as a function of time at a particle concentration of 1 μg mL^{-1}.

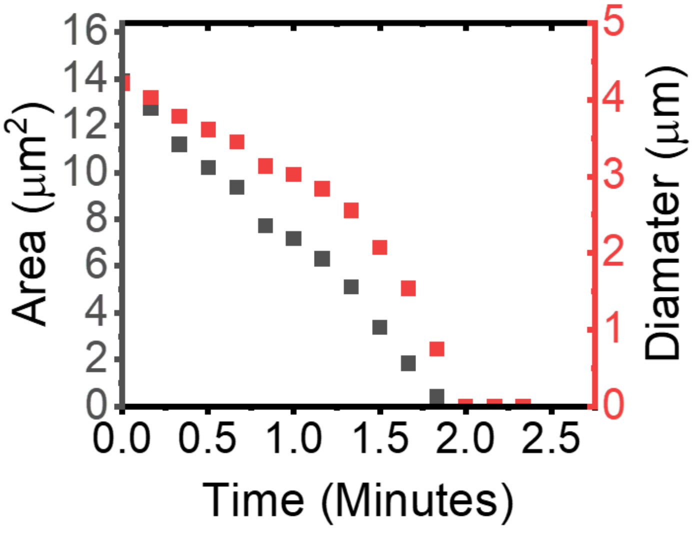

Figure S26. Measured projection area (black) and diameter (red) of an irinotecan loaded vaterite particle in 0 % human serum (pure water), plotted as a function of time at a particle concentration of 1 μg mL^{-1}.

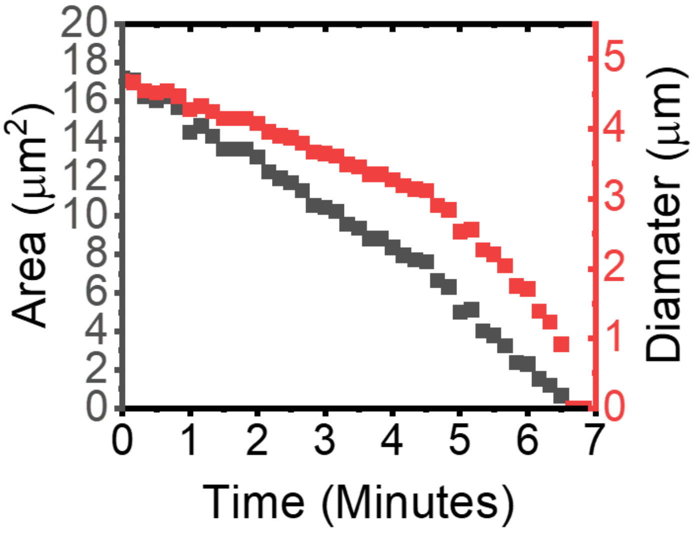

Figure S27. Measured projection area (black) and diameter (red) of an irinotecan loaded vaterite particle in 16.6 % human serum (calculated from the volumetric fraction of human serum in deionized water), plotted as a function of time at a particle concentration of 1 μg mL^{-1}.

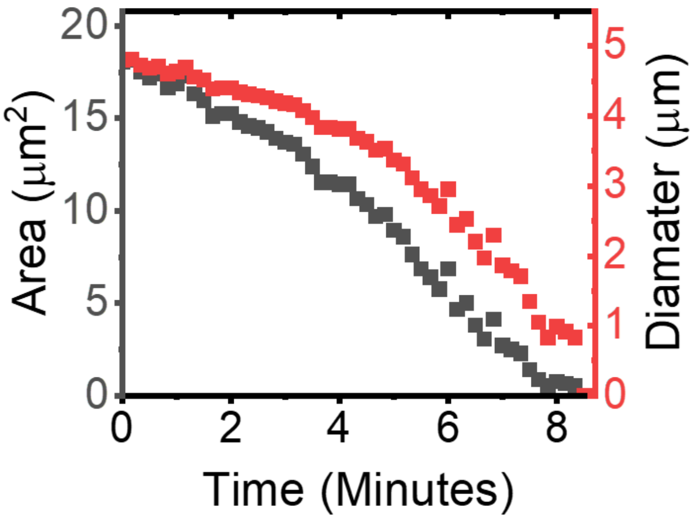

Figure S28. Measured projection area (black) and diameter (red) of an irinotecan loaded vaterite particle in 66.6 % human serum (calculated from the volumetric fraction of human serum in deionized water), plotted as a function of time at a particle concentration of 1 μg mL^{-1}.

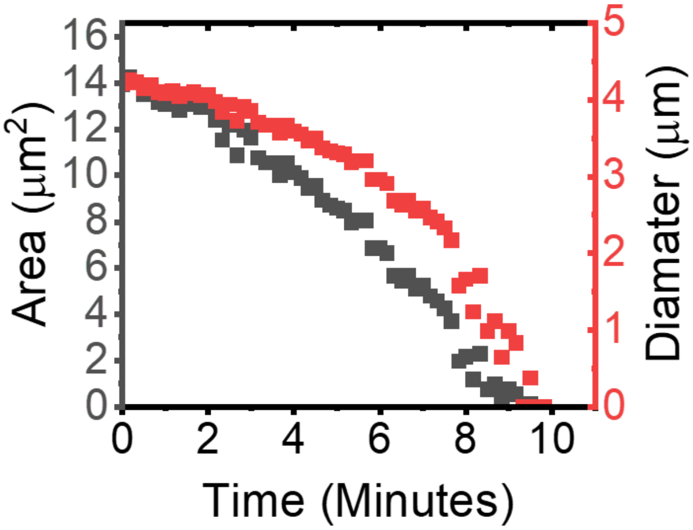

Figure S29. Measured projection area (black) and diameter (red) of an irinotecan loaded vaterite particle in 100 % human serum (pure), plotted as a function of time at a particle concentration of 1 μg mL^{-1}.

Note that the raw images from the initial study of vaterite dissolution in neat human serum showed that particles were not always stationary on the surface of the observation chamber, in contrast to their behavior in deionized water. An example image sequence of a particle dissolving in neat human serum, shown both before and after application of the auto-thresholding algorithm, is included in Figure S30. For particles that were not static throughout the full dissolution period, the dissolution rate was comparable to that observed when particles remained stationary. It was therefore concluded that particle motion on the chamber surface does not significantly affect the dissolution rate, as the rate of particle movement is low compared with the flux of ions away from the particle surface, which is a rate-determining factor under thermodynamic control.

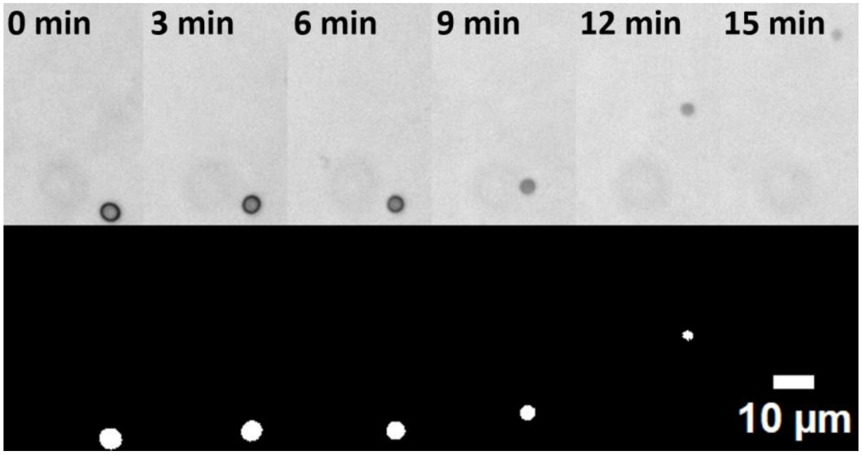

Figure S30. A series of optical microscopy images showing the dissolution of a vaterite particle in 100 % human serum at a particle concentration of 1 µg mL^{-1}. Images before (top) and after (bottom) application of the auto-thresholding algorithm are shown.

Section 6. Effect of Fe(II) on the Dissolution of Vaterite

Some of the most abundant organic ions and gases, as well as organic metabolites in the human serum metabolome are presented in Table S3.[10] Of these, Fe^{2+} was identified as a highly abundant material, at 9 mM, with a possible strong adsorption onto vaterite which can overcome complexation with proteins in human serum. It was hence considered as contributing to the observed reduction in dissolution rate caused by human serum, and the concentration dependance of this observation.

Inorganic Ions and Gases	Total Concentration (mM)
Sodium	144
Chlorine	110
Bicarbonate/carbon dioxide	36
Iron	9
Oxygen	6
Potassium	4.5
**Organic Metabolites**	**Total Concentration (mM)**
D-glucose	5
Total cholesterol	5
Melanin	5
Urea	4
ATP	3
Glyceraldehyde	1.5

Table S3. Table of abundant inorganic ions and gases, and organic metabolites, present in the human serum metabolome.[10]

The effect of Fe^{2+} on dissolution rate in deionized water was studied by suspending vaterite particles in different concentration of $FeCl_2$, using the standard dissolution in deionized water experimental setup and method. Representative raw images of example particles dissolving at 3, 20, and 80 µM $FeSO_4$ are inlcuded in Figure S31 to S33. As the concentration was increased,

before any reduction in rate could be observed, the precipitation of another material, likely $FeCO_3$, was observed.

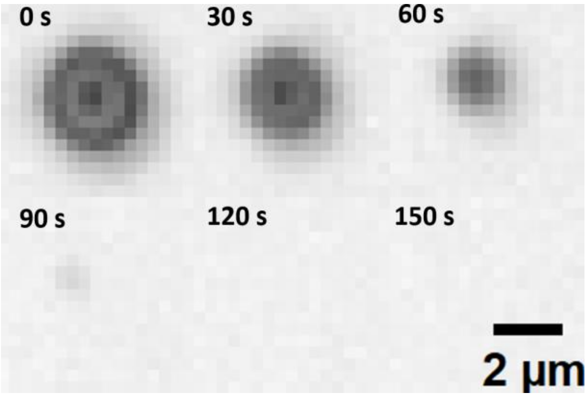

Figure S31. A series of optical microscopy images showing the dissolution of a vaterite particle in 3 µM $FeCl_2$ at a particle concentration of 1 µg mL^{-1}.

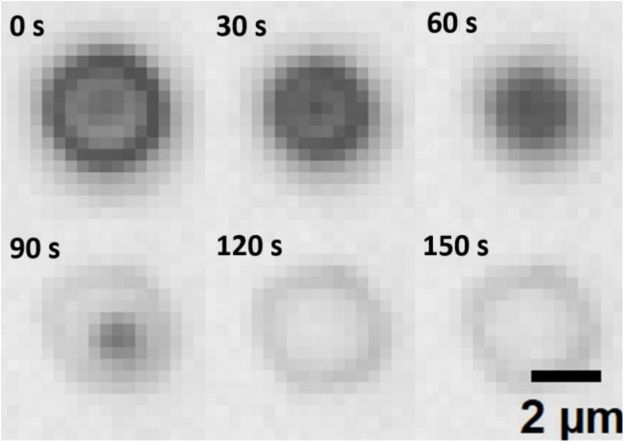

Figure S32. A series of optical microscopy images showing the dissolution of a vaterite particle in 20 µM $FeCl_2$ at a particle concentration of 1 µg mL^{-1}.

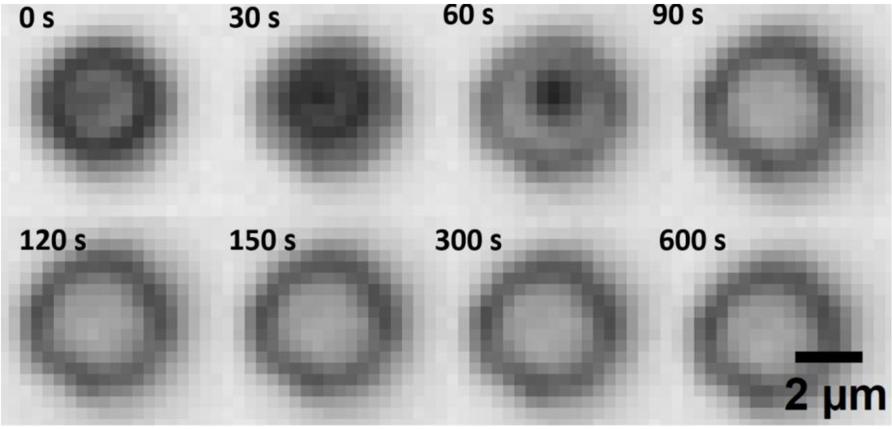

Figure S33. A series of optical microscopy images showing the dissolution of a vaterite particle in 80 μM $FeCl_2$ at a particle concentration of 1 μg mL^{-1}.

Further, at 80 μM $FeSO_4$ the dissolution rate of the vaterite material can still be observed as shrinking of the darker material of the core in Figure S33, which can then be converted to a dissolution rate by manually measuring its area at different times. The average dissolution rate for five particles in 3 and 20 μM $FeSO_4$ is similar to that in DI water, while the rate in 80 μM $FeSO_4$ is more variable but generally faster than in DI water. This is likely due to the strong driving force for $FeCO_3$ formation. Given the observed precipitation of a distinct phase at higher Fe(II) concentrations, without any corresponding inhibition of vaterite dissolution, it is unlikely that Fe^{2+} contributes to the rate inhibition observed in the human serum experiments.

References

(1) di Nunzio, M. R.; Douhal, Y.; Organero, J. A.; Douhal, A. Structural and Photodynamic Properties of the Anti-Cancer Drug Irinotecan in Aqueous Solutions of Different PHs. *Physical Chemistry Chemical Physics* **2018**, *20* (20), 14182–14191. https://doi.org/10.1039/C8CP01134F.

(2) Wu, J.-L.; Wang, C.-Q.; Zhuo, R.-X.; Cheng, S.-X. Multi-Drug Delivery System Based on Alginate/Calcium Carbonate Hybrid Nanoparticles for Combination Chemotherapy. *Colloids Surf. B Biointerfaces* **2014**, *123*, 498–505. https://doi.org/10.1016/j.colsurfb.2014.09.047.

(3) Khudina, E. A.; Tsyupka, D. V.; Drozd, D. D.; Goryacheva, I. Yu.; Goryacheva, O. A. Cytostatic Drug Mitoxantrone Delivery Platform Based on Porous Vaterite Particles: Synthesis, Characterization and Release. *Mater. Today Commun.* **2024**, *40*, 109451. https://doi.org/10.1016/j.mtcomm.2024.109451.

(4) Dunuweera, S. P.; Rajapakse, R. M. G. Encapsulation of Anticancer Drug Cisplatin in Vaterite Polymorph of Calcium Carbonate Nanoparticles for Targeted Delivery and Slow Release. *Biomed. Phys. Eng. Express* **2017**, *4* (1), 015017. https://doi.org/10.1088/2057-1976/aa9719.

(5) Qiu, N.; Yin, H.; Ji, B.; Klauke, N.; Glidle, A.; Zhang, Y.; Song, H.; Cai, L.; Ma, L.; Wang, G.; Chen, L.; Wang, W. Calcium Carbonate Microspheres as Carriers for the Anticancer Drug Camptothecin. *Materials Science and Engineering: C* **2012**, *32* (8), 2634–2640. https://doi.org/10.1016/j.msec.2012.08.026.

(6) Elbaz, N. M.; Owen, A.; Rannard, S.; McDonald, T. O. Controlled Synthesis of Calcium Carbonate Nanoparticles and Stimuli-Responsive Multi-Layered Nanocapsules for Oral Drug Delivery. *Int. J. Pharm.* **2020**, *574*, 118866. https://doi.org/10.1016/j.ijpharm.2019.118866.

(7) Kontoyannis, C. G.; Vagenas, N. V. Calcium Carbonate Phase Analysis Using XRD and FT-Raman Spectroscopy. *Analyst* **2000**, *125* (2), 251–255. https://doi.org/10.1039/a908609i.

(8) Milner, M. P.; Yang, M.; Compton, R. G. Vaterite Dissolution: Mechanism and Kinetics. *J. Phys. Chem. C* **2024**, *128* (25), 10388–10396. https://doi.org/10.1021/acs.jpcc.4c02074.

(9) Milner, M. P.; Yang, M.; Compton, R. G. Correction to Vaterite Dissolution: Mechanism and Kinetics. *J. Phys. Chem. C* **2024**, *128* (40), 17196–17196. https://doi.org/10.1021/acs.jpcc.4c06304.

(10) Psychogios, N.; Hau, D. D.; Peng, J.; Guo, A. C.; Mandal, R.; Bouatra, S.; Sinelnikov, I.; Krishnamurthy, R.; Eisner, R.; Gautam, B.; Young, N.; Xia, J.; Knox, C.; Dong, E.; Huang, P.; Hollander, Z.; Pedersen, T. L.; Smith, S. R.; Bamforth, F.; Greiner, R.; McManus, B.; Newman, J. W.; Goodfriend, T.; Wishart, D. S. The Human Serum Metabolome. *PLoS One* **2011**, *6* (2), e16957. https://doi.org/10.1371/journal.pone.0016957.
